# Pericyte‐secreted IGF2 promotes breast cancer brain metastasis formation

**DOI:** 10.1002/1878-0261.12752

**Published:** 2020-06-26

**Authors:** Kinga Molnár, Ádám Mészáros, Csilla Fazakas, Mihály Kozma, Fanni Győri, Zita Reisz, László Tiszlavicz, Attila E. Farkas, Ádám Nyúl‐Tóth, János Haskó, István A. Krizbai, Imola Wilhelm

**Affiliations:** ^1^ Institute of Biophysics Biological Research Centre Szeged Hungary; ^2^ Theoretical Medicine Doctoral School University of Szeged Szeged Hungary; ^3^ Doctoral School of Biology University of Szeged Szeged Hungary; ^4^ Department of Physiology, Anatomy and Neuroscience University of Szeged Szeged Hungary; ^5^ Department of Pathology University of Szeged Szeged Hungary; ^6^ Vascular Cognitive Impairment and Neurodegeneration Program Department of Biochemistry and Molecular Biology Reynolds Oklahoma Center on Aging/Oklahoma Center for Geroscience University of Oklahoma Health Sciences Center Oklahoma City OK USA; ^7^ Institute of Life Sciences Vasile Goldiş Western University of Arad Arad Romania

**Keywords:** brain metastasis, cerebral pericyte, insulin‐like growth factor 2, picropodophyllin, triple‐negative breast cancer

## Abstract

Brain metastases are life‐threatening complications of triple‐negative breast cancer, melanoma, and a few other tumor types. Poor outcome of cerebral secondary tumors largely depends on the microenvironment formed by cells of the neurovascular unit, among which pericytes are the least characterized. By using *in vivo* and *in vitro* techniques and human samples, here we show that pericytes play crucial role in the development of metastatic brain tumors by directly influencing key steps of the development of the disease. Brain pericytes had a prompt chemoattractant effect on breast cancer cells and established direct contacts with them. By secreting high amounts of extracellular matrix proteins, pericytes enhanced adhesion of both melanoma and triple‐negative cancer cells, which might be particularly important in the exclusive perivascular growth of these tumor cells. In addition, pericytes secreted insulin‐like growth factor 2 (IGF2), which had a very significant pro‐proliferative effect on mammary carcinoma, but not on melanoma cells. By inhibiting IGF2 signaling using silencing or picropodophyllin (PPP), we could block the proliferation‐increasing effect of pericytes on breast cancer cells. Administration of PPP (a blood–brain barrier‐permeable substance) significantly decreased the size of brain tumors in mice inoculated with triple‐negative breast cancer cells. Taken together, our results indicate that brain pericytes have significant pro‐metastatic features, especially in breast cancer. Our study underlines the importance of targeting pericytes and the IGF axis as potential strategies in brain metastatic diseases.

## Graphical abstract

Here, we show that brain pericytes have significant pro‐metastatic features, especially in breast cancer. Brain pericytes enhanced adhesion of tumor cells to support their exclusive perivascular growth. Pericytes also secreted IGF2 to promote proliferation of cancer cells. By blocking the IGF axis, the proliferation increasing effect of brain pericytes could be inhibited both *in vitro* and *in vivo*.

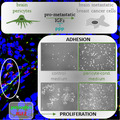

**Abbreviations**
BBB: blood–brain barrier
CEC: cerebral endothelial cell
CNS: central nervous system
EGFP: enhanced green fluorescent protein
EmGFP: emerald green fluorescent protein
EMT: epithelial‐to‐mesenchymal transition
EV: extracellular vesicle
FAK: focal adhesion kinase
HA: human astrocyte
HBVP: human brain vascular pericyte
IGF: insulin‐like growth factor
IGF1R: type 1 insulin‐like growth factor receptor
NVU: neurovascular unit
P/S: penicillin/streptomycin
PDGFRβ: platelet‐derived growth factor receptor β
PM: pericyte medium
PPP: picropodophyllin
RIPA: radioimmunoprecipitation assay buffer
TNBC: triple‐negative breast cancer

## 1 Introduction

Cerebral metastases are frequent and dismal complications of a few tumor cell types, including lung cancer, breast cancer, and melanoma. In breast cancer, which is the second most frequent cause of central nervous system (CNS) metastases, brain lesions primarily occur in triple‐negative (estrogen receptor‐, progesterone receptor‐, and Her2‐negative) and Her2/neu‐overexpressing subtypes. Triple‐negative breast cancer (TNBC) has a particularly poor prognosis, many patients developing and eventually dying of brain metastasis [1]. Melanoma, the third most common brain metastatic tumor, represents the highest risk to spread to the CNS, and brain metastases are a leading cause of death from this disease [2].

Presence of the blood–brain barrier (BBB) [3], tumor heterogeneity and plasticity [4], and unique molecular features of brain metastatic cells [5] all contribute to the poor outcome of cerebral secondary tumors. The most important unique aspects of brain metastasis development are linked to the neurovascular unit (NVU), that is, cerebral endothelial cells (CECs), pericytes, glial cells, and neurons, with whom tumor cells form complex interactions [6]. These interactions have been extensively studied, except for pericytes.

Brain pericytes are mural cells located in the duplication of the basement membrane of microvessels, coming in close contact with cerebral endothelial and glial cells. They may act as regulators of vessel formation and stabilization [7, 8], BBB permeability [9, 10] and—although still debated—cerebral blood flow [11, 12]. Pericytes play a crucial role in various neurological diseases, including ischemia, traumatic injury, neurodegenerative and neuro‐immune conditions, and also glioblastoma [13]. However, interactions of brain pericytes with metastatic tumor cells are largely unexplored, although pericytes have recently emerged as important players in creating the premetastatic niche in peripheral organs [14]. Since microvessels of the CNS have the highest pericyte coverage in the organism [15], we hypothesized that pericyte‐metastatic cell interactions might be particularly critical in the brain.

## 2 Materials and methods

### 2.1 Cell culture

#### 2.1.1 Culture of human NVU cells

Human brain vascular pericytes (abbreviated as HBVP; obtained from ScienCell Research Laboratories, Carlsbad, CA, USA) were cultured on poly‐l‐lysine‐coated dishes in Pericyte Medium (PM; ScienCell Research Laboratories) supplemented with 5% FBS (Sigma‐Aldrich, St. Louis, MO, USA) and penicillin/streptomycin solution (P/S; ScienCell Research Laboratories) and used between passage numbers 2 and 6. Human astrocytes (HA; ScienCell Research Laboratories) were grown on poly‐l‐lysine‐coated dishes in astrocyte medium (AM) supplemented with 5% FBS and P/S (all from ScienCell Research Laboratories) and used between passage numbers 2 and 4. Human microvascular cerebral endothelial cells (hCMEC/D3, shortly D3) were cultured on rat‐tail collagen‐coated dishes in endothelial cell basal medium‐2 (EBM‐2) with EGM‐2MV kit including supplements and 2% FBS (all from Lonza, Basel, Switzerland) and used between passage numbers 30 and 40.

#### 2.1.2 Isolation and culture of mouse NVU cells

Venus‐YFP‐expressing primary mouse brain endothelial cells were isolated from 6‐ to 8‐week‐old FVB/Ant:TgCAG‐yfp_sb #27 mice, as described previously [16]. Murine pericytes were collected in parallel with endothelial cells after the first enzymatic digestion. Pericytes were seeded onto rat‐tail collagen‐coated dishes and kept in PM supplemented with 5% FBS (Sigma‐Aldrich) and P/S solution. Mouse pericytes were used for experiments between passage numbers 5 and 7. Primary mouse astrocytes were isolated from brains of 1‐ to 3‐day‐old Balb/c mouse pups by mechanical trituration. Astrocytes were seeded onto poly‐l‐lysine‐coated dishes and kept in low‐glucose Dulbecco's modified Eagle's medium (DMEM; Thermo Fisher Scientific, Waltham, MA, USA) supplemented with 10% FBS (Sigma‐Aldrich).

#### 2.1.3 Culture of tumor cells

MDA‐MB‐231 human triple‐negative breast cancer cells (shortly MDA), enhanced green fluorescent protein (EGFP)‐expressing MDA‐MB‐231 cells (shortly MDA‐EGFP); mouse triple‐negative mammary carcinoma cells 4T1, 4T1‐tdTomato and 4T1‐EmGFP (expressing emerald GFP); A2058 human and B16/F10 (abbreviated as B16) murine melanoma cells were cultured as described previously [17]. A2058‐EGFP cells were obtained by transfection with the pEGFP‐C1 plasmid using TurboFect reagent (Thermo Fisher Scientific) and selection with 500 μg·mL^−1^ G418 (Thermo Fisher Scientific).

All cell lines were regularly tested for mycoplasma contamination using the MycoAlert Mycoplasma Detection Kit (Lonza). Only mycoplasma‐negative cultures were used for experiments.

### 2.2 Preparation of conditioned media

Human and mouse cells were grown until 90% confluency in 6‐cm culture dishes. Growth medium was replaced with HBVP complete medium (PM + 5% FBS + P/S) and left for 2 days. Nonconditioned complete medium was used as negative control. All media were filtered through 0.2‐µm pore size syringe filters to remove cells and debris.

Extracellular vesicles (EVs) were depleted from HBVP control and conditioned media by using a serial centrifugation protocol: 700 ***g***, 5 min; 1000 ***g***, 8 min; 10 000 ***g***, 30 min and 150 000 ***g***, 90 min. Media were collected and filtered through 0.1‐µm pore size syringe filters.

### 2.3 Experimental animal surgeries

All mice were housed and treated in accordance with widely accepted standards, and the protocols were approved by the institutional care and the Regional Animal Health and Food Control Station of Csongrád County (permit number: XVI./764/2018). Surgeries were carried out on 6‐ to 10‐week‐old female FVB/Ant:TgCAG‐yfp_sb #27 mice (obtained from Institute of Experimental Medicine, Budapest, Hungary), as described previously [16]. For *ex vivo* experiments, 6‐ to 10‐week‐old female mice received 10^6^ 4T1‐tdTomato cells in 100 µL into the right common carotid artery. For testing the effect of picropodophyllin (PPP) on tumor growth *in vivo*, animals were inoculated with 3 × 10^6^ 4T1‐tdTomato cells/200 µL by intracardiac injection. The well‐being of the animals was monitored daily during the postoperative period. No infection or wound dehiscence was detected, neither any signs of chronic pain. Maximum 5% of weight loss was observed, and the operation‐induced mortality rate was 2–3%. On days 5 and 6 postinjection, mice received 40 mg·kg^−1^ PPP (Alfa Aesar, Thermo Fisher Scientific) in corn oil or vehicle only, intraperitoneally. On day 8, mice were transcardially perfused. Coronary brain sections were prepared from all animals from parietal cortical regions, and the same volumes were used for imaging with a Leica SP5 microscope (Leica Biosystems, Wetzlar, Germany). Tumor‐covered areas in control and PPP‐treated animals were measured with fiji software using a custom‐made macro [18]. 

### 2.4 Immunofluorescence

Human triple‐negative breast cancer brain metastasis and control brain samples were acquired from the Department of Pathology, University of Szeged. Human samples were collected in accordance with the permission of the Human Investigation Review Board, University of Szeged (permit number: EMLOSEB001, issued on January 31, 2017), in agreement with the Declaration of Helsinki of the World Medical Association. Surgical samples were fixed in PFA and embedded into paraffin blocks. Five‐micrometer‐thick slices were used for immunofluorescence staining. After deparaffinization in xylol, samples were rehydrated through a descending series of alcohol to water. Antigen retrieval was performed by boiling in 10 mm sodium citrate pH = 6 for 15 min.

After fixation, whole mouse brains were mounted onto a freezing microtome (Reichert‐Jung, Leica Biosystems) and 20‐µm‐thick coronal brain sections were cut, which were kept in PBS with 0.05% sodium azide until further processing. Antigen retrieval was performed by incubating the sections at 80 °C for 30 min in 10 mm sodium citrate pH = 6.

All sections were permeabilized in 0.5% Triton X‐100 (Sigma‐Aldrich) for 20 min, then blocked with 3% BSA (VWR International, Radnor, PA, USA) in PBS containing 0.5% Triton X‐100 for 1 h. Primary antibodies (Table S1) were diluted in 1% BSA with 0.5% Triton X‐100, and sections were incubated overnight at 4 °C on an orbital shaker. Slides were extensively washed in PBS. Alexa Fluor 488‐labeled anti‐rabbit, Alexa Fluor 594‐labeled anti‐mouse, Alexa Fluor 647‐labeled anti‐goat IgG (Thermo Fisher Scientific), and STAR RED‐labeled anti‐rabbit IgG (Abberior, Göttingen, Germany) were used as secondary antibodies in a dilution of 1 : 300 in PBS for 60 min at room temperature in the dark. Sections were washed, counterstained with a nuclear dye (Hoechst 33342; Sigma‐Aldrich) for 10 min, washed again with PBS, rinsed in water, and mounted in FluoroMount‐G (SouthernBiotech, Birmingham, AL, USA).

Samples were analyzed using a Leica SP5 confocal laser scanning microscope.

### 2.5 Adhesion experiments

#### 2.5.1 Adhesion in co‐culture

HBVP cells were stained with CellTracker™ Red CMTPX Dye (Thermo Fisher Scientific), extensively washed, and mixed with MDA‐EGFP cells in a ratio of 1 : 1. Mixed culture was seeded onto poly‐l‐lysine‐coated dishes in complete HBVP medium and left for 3 days.

#### 2.5.2 Cell‐to‐cell adhesion

D3, HBVP, or HA cells were seeded into 12‐well plates (noncoated or coated with poly‐l‐lysine or rat‐tail collagen) in a density of 2 × 10^4^ cells/well. After 24 h, 1.5 × 10^4^ cells/well MDA‐EGFP or A2058‐EGFP were seeded onto the cells and incubated for 24 h. Phase‐contrast and fluorescence images were taken with a Nikon Eclipse TE2000U (Nikon, Tokyo, Japan) microscope connected to a digital camera (Spot RT KE, Diagnostic Instruments, Sterling Heights, MI, USA). Attached fluorescent cells were counted using fiji software [18].

#### 2.5.3 Cell‐to‐surface adhesion

5 × 10^5^ tumor cells were seeded into 6‐well plates in pericyte‐conditioned or control media in the presence or absence of Src inhibitors (Selleck Chemicals, Munich, Germany) and left for 20 min (A2058, 4T1 and B16) or 120 min (MDA). Floating cells were collected by centrifugation, and phase‐contrast images were taken from attached cells. Efficiency of cell adhesion was determined by counting the adherent cells. For further western blot analysis, attached and floating cells were mixed together in radioimmunoprecipitation assay buffer (RIPA).

In another setup, 6‐well plates were pretreated with endothelial cell‐, pericyte‐, or astrocyte‐conditioned media for 24 h. Conditioned media were removed, and 5 × 10^5^ tumor cells were seeded into the pretreated wells in control medium.

### 2.6 Migration assays

#### 2.6.1 Short‐distance migration assay (wound healing)

3 × 10^4^ MDA cells and 1.5 × 10^4^ HBVP or 3 × 10^4^ D3 cells were seeded into rat‐tail collagen‐coated silicone 3‐well culture inserts (Ibidi, Graefelfing, Germany). After reaching confluency, the insert was removed and culture medium was changed to Leibovitz's L‐15 medium (Sigma‐Aldrich) supplemented with 1% FBS. Migration of cells was monitored for 24 h in phase‐contrast.

#### 2.6.2 Long‐distance migration assay

3 × 10^4^ HBVP cells and 6 × 10^4^ MDA‐EGFP and D3 cells were seeded into every second well of a rat‐tail collagen‐coated silicone 12‐well culture insert (Ibidi). After reaching confluency, the insert was removed. After 5 days, phase‐contrast and fluorescence images were taken from the whole area and an overview image was generated.

### 2.7 Proliferation assays

#### 2.7.1 Phase‐contrast imaging and cell number counting

10^4^ tumor cells/well were plated into 6‐well plates, and fresh medium was given every other day. For cell counting, phase‐contrast images were taken every day. After 4 days, cells were collected in RIPA buffer for western blot analysis.

#### 2.7.2 Real‐time impedance monitoring

10^4^ 4T1 or B16/F10 cells were seeded into E‐plates in control or pericyte‐conditioned medium with or without 100 nm PPP. Fresh medium was provided after 48 h. Electrical impedance was measured in real‐time using the xCELLigence system (ACEA Biosciences, San Diego, CA, USA).

### 2.8 Methanol–chloroform precipitation and western blot

Conditioned media were centrifuged to seed cell debris, and an equal volume of methanol and 1/4 volume of chloroform were added. Samples were vortexed, incubated for 5 min on ice, and centrifuged at 10 000 ***g*** for 5 min at 4 °C. After phase separation, aqueous phase was removed and protein samples were washed with ice‐cold methanol. Samples were vortexed and centrifuged again, supernatants were discarded, and protein pellets were air‐dried. Pellets were reconstituted in 2× Laemmli buffer and heated up to 95 °C for 5 min.

Cells were lysed in RIPA buffer. Protein concentration was determined by using bicinchoninic acid assay (Thermo Fisher Scientific). Laemmli buffer was added to the samples and incubated at 95 °C for 5 min. Samples were electrophoresed using standard denaturing SDS/PAGE and blotted on polyvinylidene difluoride (0.2 μm pore size; Bio‐Rad, Hercules, CA, USA, or 0.45 μm pore size; BioTrace, Pall Corporations, Port Washington, NY, USA) or nitrocellulose membranes (0.2 μm pore size; Bio‐Rad). After blocking with 3% BSA or 5% nonfat milk in Tris‐buffered saline with 0.1% Tween‐20 (TBS‐T), membranes were incubated with primary antibodies (Table S1) overnight at 4 °C. Blots were washed in TBS‐T three times for 10 min, incubated for 1 h in horseradish peroxidase‐conjugated anti‐rabbit IgG or anti‐mouse IgG secondary antibodies (Jackson ImmunoResearch, Cambridgeshire, UK) diluted to 1 : 3000 in TBS‐T, and then washed again in TBS‐T. Immunoreaction was visualized with Clarity Chemiluminescence Substrate (Bio‐Rad) in a ChemiDoc MP System (Bio‐Rad). Densitometry analysis was performed with the image lab Software, version 5.2 (Bio‐Rad).

### 2.9 RNA isolation and real‐time polymerase chain reaction

Total RNA was isolated using TRIfast reagent (VWR International). Maxima First Strand cDNA Synthesis Kit (Thermo Fisher Scientific) was used to transcribe RNA into cDNA. Amplification was performed using iTaq™ Universal SYBR^®^ Green Supermix (Bio‐Rad) or Luminaris Color HiGreen qPCR Master Mix kit (Thermo Fisher Scientific) on a Bio‐Rad iQ5 instrument under the following conditions: 40 cycles of 95 °C for 15 s, 56–60 °C for 30 s, 72 °C for 30 s using primers detailed in Table S2.

### 2.10 Enzyme‐linked immunosorbent assay

After collection of conditioned media, cells were trypsinized and counted in a hemocytometer. Conditioned media were centrifuged at 1000 ***g*** for 15 min at 4 °C. Insulin‐like growth factor 1 (IGF1) and IGF2 contents of conditioned media were measured using commercial ELISA kits (CSB‐E04580h and CSB‐E04583h, respectively; Cusabio, Wuhan, China) following the manufacturer's instructions.

### 2.11 Silencing of Igf2 gene

Stealth™ siRNA duplex oligoribonucleotides were designed using Invitrogen BLOCK‐iT™ RNAi designer and purchased from Thermo Fisher Scientific. The following sequences were used: sense, 5′‐UCGAUGCUGGUGCUUCUCACCUUCU‐3′ and antisense, 5′‐AGAAGGUGAGAAGCACCAGCAUCGA‐3′. Cell transfection was performed with Lipofectamine 2000, as previously described [19].

### 2.12 Statistical analysis

Student's *t*‐test and analysis of variance (ANOVA) were performed using the Excel 2016 Data Analysis plugin. For *post hoc* tests, we applied sigmaplot version 12.3 (Systat Software Inc., Chicago, IL, USA).

## 3 Results

### 3.1 Direct interaction of brain metastatic tumor cells with pericytes *in vivo* and *in vitro*

**Fig. 1:**
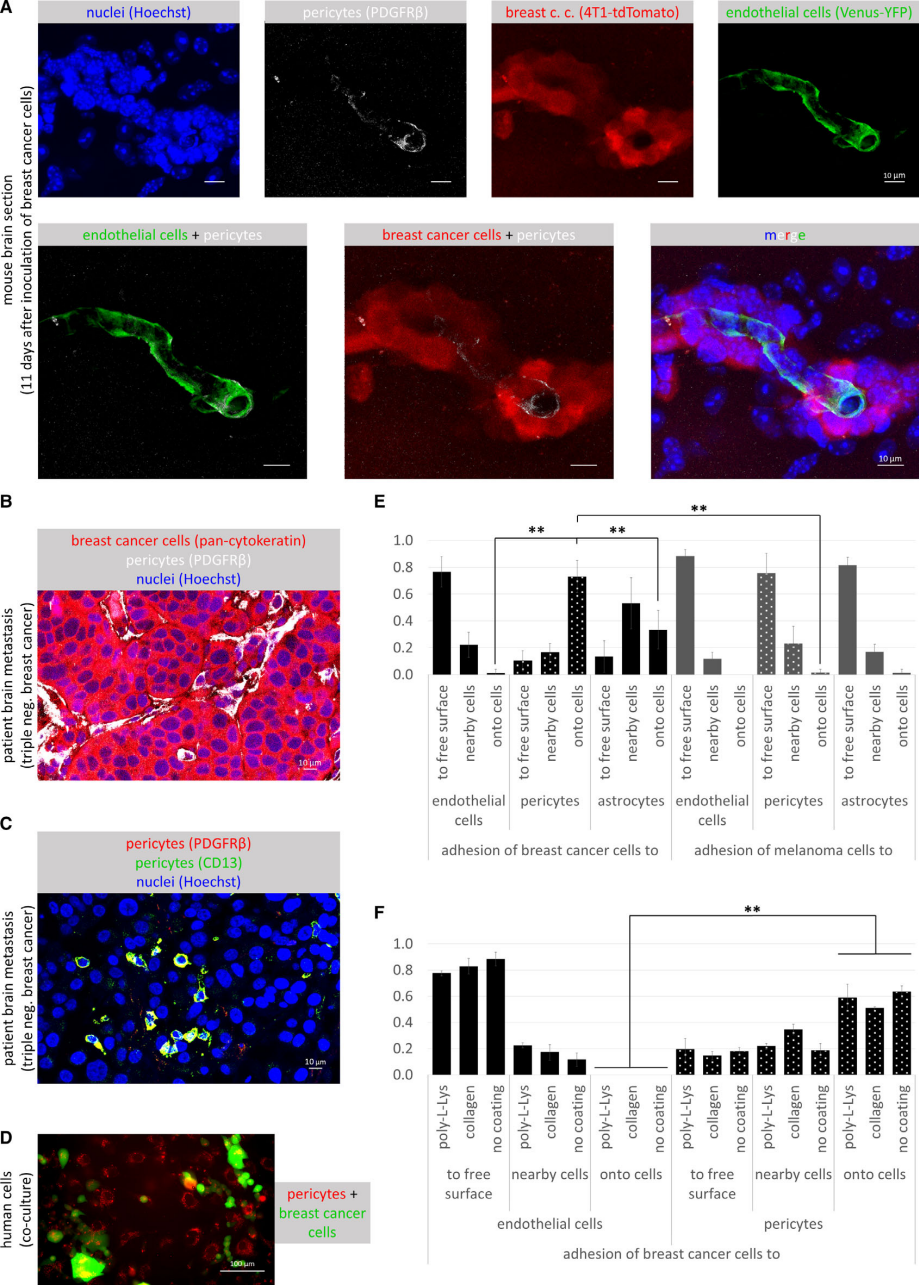
Direct interactions of pericytes with tumor cells. (A) Brain sections of FVB/Ant:TgCAG‐yfp mice 11 days after injection of 4T1‐tdTomato cells showing PDGFRβ‐positive pericytes localized along capillaries inside the tumor mass. Scale bars = 10 µm. (B) Human triple‐negative brain metastatic tissue with PDGFRβ‐positive pericytes in the stroma. Scale bar = 10 µm. (C) Human triple‐negative brain metastatic tissue with PDGFRβ (red) and CD13 (green) double‐positive (yellow) pericyte‐like cells scattered among tumor cells. Scale bar = 10 µm. (D) Co‐culture of CellTracker™ Red‐stained human pericytes (HBVP cells) and human triple‐negative breast cancer (MDA‐EGFP) cells 3 days after seeding. Scale bar = 100 µm. (E) Quantification of tumor cell adhesion 24 h after seeding onto brain cells cultured in poly‐l‐lysine‐coated dishes. Breast cancer cells: MDA‐EGFP, melanoma cells: A2058‐EGFP, endothelial cells: D3, pericytes: HBVP, astrocytes: HA. *N* = 5, average ± SD, **P < 0.01 (ANOVA and Bonferroni's *post hoc* test). (F) Comparison of human breast cancer cell (MDA‐EGFP) adhesion to D3 and HBVP cells. *N* = 3, average ± SD, **P < 0.01 (ANOVA and Bonferroni's *post hoc* test).

We have previously shown that TNBC cells proliferate along capillaries in the mouse brain, co‐opting the endothelium, but expelling astrocytes and astrocyte end‐feet to the surface of the tumor [16]. Since tumor co‐opted endothelial cells maintained tight junctions in the absence of astrocyte end‐foot coverage, we investigated whether pericytes—another cell type involved in the induction of barrier properties of CECs [9, 10]—remained in contact with the vessels, as previously described in a different metastatic model [20]. Indeed, we found that PDGFRβ (platelet‐derived growth factor receptor β)‐positive pericytes were localized to capillaries inside metastatic lesions in the mouse brain (Fig. 1A). In human TNBC brain metastases, PDGFRβ‐positive perivascular cells were found in the stroma (Fig. 1B), in single or multiple layers, as previously shown [21]. In addition, we have also detected single pericyte‐like cells expressing the specific markers PDGFRβ and CD13, scattered among the tumor cells, especially in less cell‐dense areas, probably in the proximity of necrotic zones (Fig. 1C).

All these data prompted us to hypothesize that direct interactions between tumor cells and pericytes might influence brain metastasis development. Therefore, we first modeled contacts between metastatic cells and pericytes using an *in vitro* setup, by plating the tumor cells onto sparse cultures of brain pericytes and other cells of the NVU. Breast cancer cells preferentially gathered onto the top of pericytes, avoiding the cell‐free culture surface (Fig. 1D,E), and this was independent of the coating of the culture dish (Fig. 1F). In contrast, when breast cancer cells were co‐cultured with CECs, they seldom adhered onto endothelial cells, but rather into gaps among them. In co‐culture with astrocytes, direct contacts of the tumor cells were more frequent than with endothelial cells, but less preferred than interaction with pericytes (Fig. 1E, Fig. S1). In contrast to breast cancer cells, the highest number of melanoma cells attached to free surfaces among the cells, independent of the cell type they were co‐cultured with (Fig. 1E, Fig. S1).

**Fig. 2:**
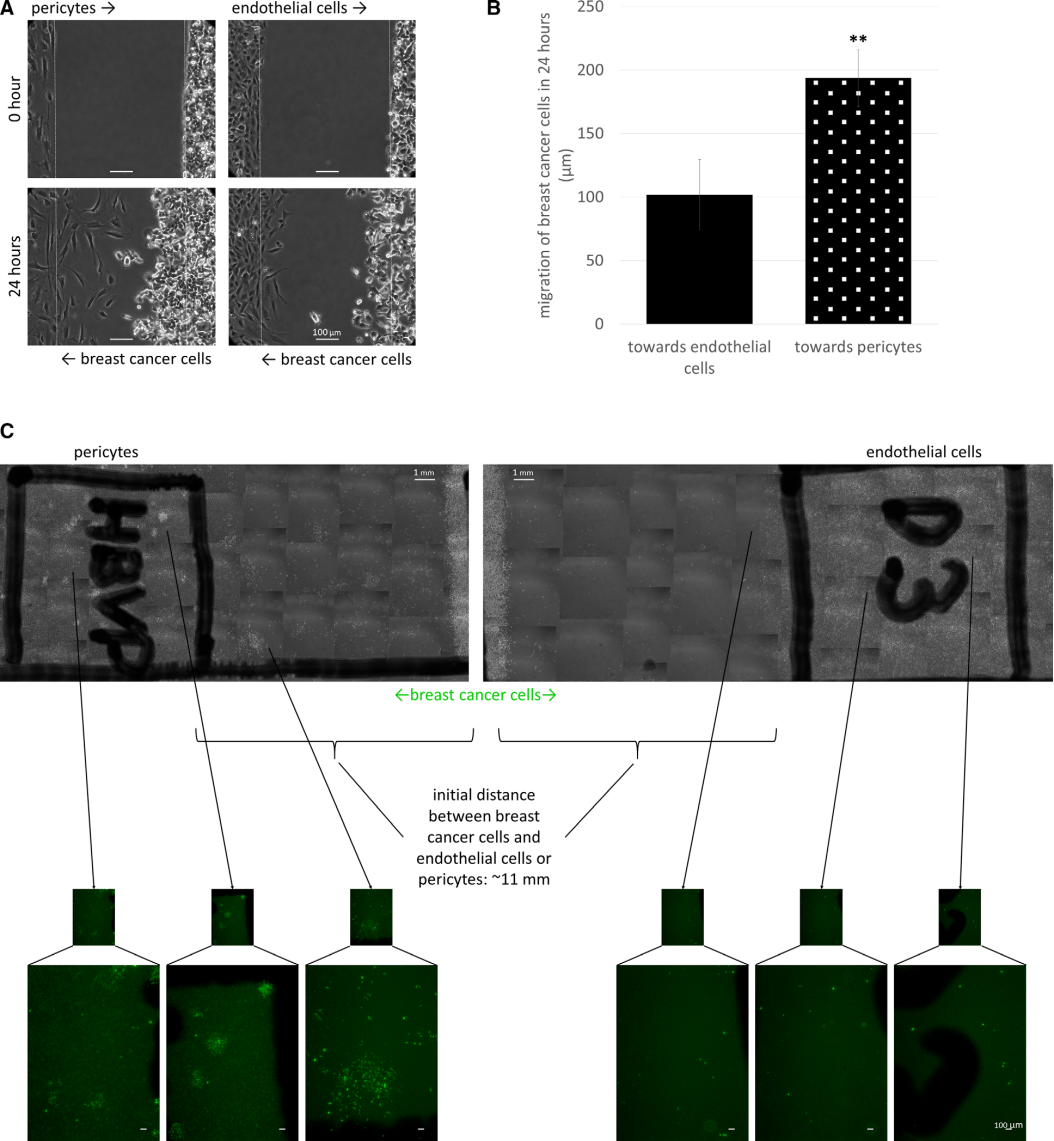
Migration of breast cancer cells toward brain pericytes. (A) Representative phase‐contrast images of pericyte‐breast cancer cell and brain endothelial‐breast cancer cell migration (short‐distance invasion assay—described in Materials and methods). Scale bar = 100 µm. (B) Quantitative analysis of the short‐distance wound assays at 24 h. *N* = 5, average ± SD, **P < 0.01 (Student's *t*‐test). (C) Phase‐contrast and fluorescence (green insets) images of HBVP, D3, and MDA‐EGFP cells after 5 days of migration in the long‐distance invasion assay. Scale bars = 1 mm (top panels) and 100 µm (bottom panels).

As a next step, we aimed to explore whether breast cancer cells could actively migrate in the direction of brain pericytes. Carcinoma cells readily migrated toward pericytes, covering a significantly larger distance on their way to brain pericytes than to endothelial cells (Fig. 2A,B). When leaving a much longer distance (more than 1 cm) between tumor cells and two different brain cells (endothelial cells and pericytes, respectively), breast carcinoma cells preferentially migrated in the direction of pericytes. After a few days, several breast cancer cells were detected in the initially cell‐free area between tumor cells and pericytes and large breast cancer cell colonies were formed among pericytes. On the other hand, only a few scattered mammary carcinoma cells were observed in the direction of and among endothelial cells (Fig. 2C). These results indicate that pericytes might communicate with tumor cells through secreted factors. Therefore, we conditioned culture medium on brain pericytes to characterize in details its effects on neoplastic cells.

### 3.2 Effects of pericytes on tumor cell adhesion

**Fig. 3:**
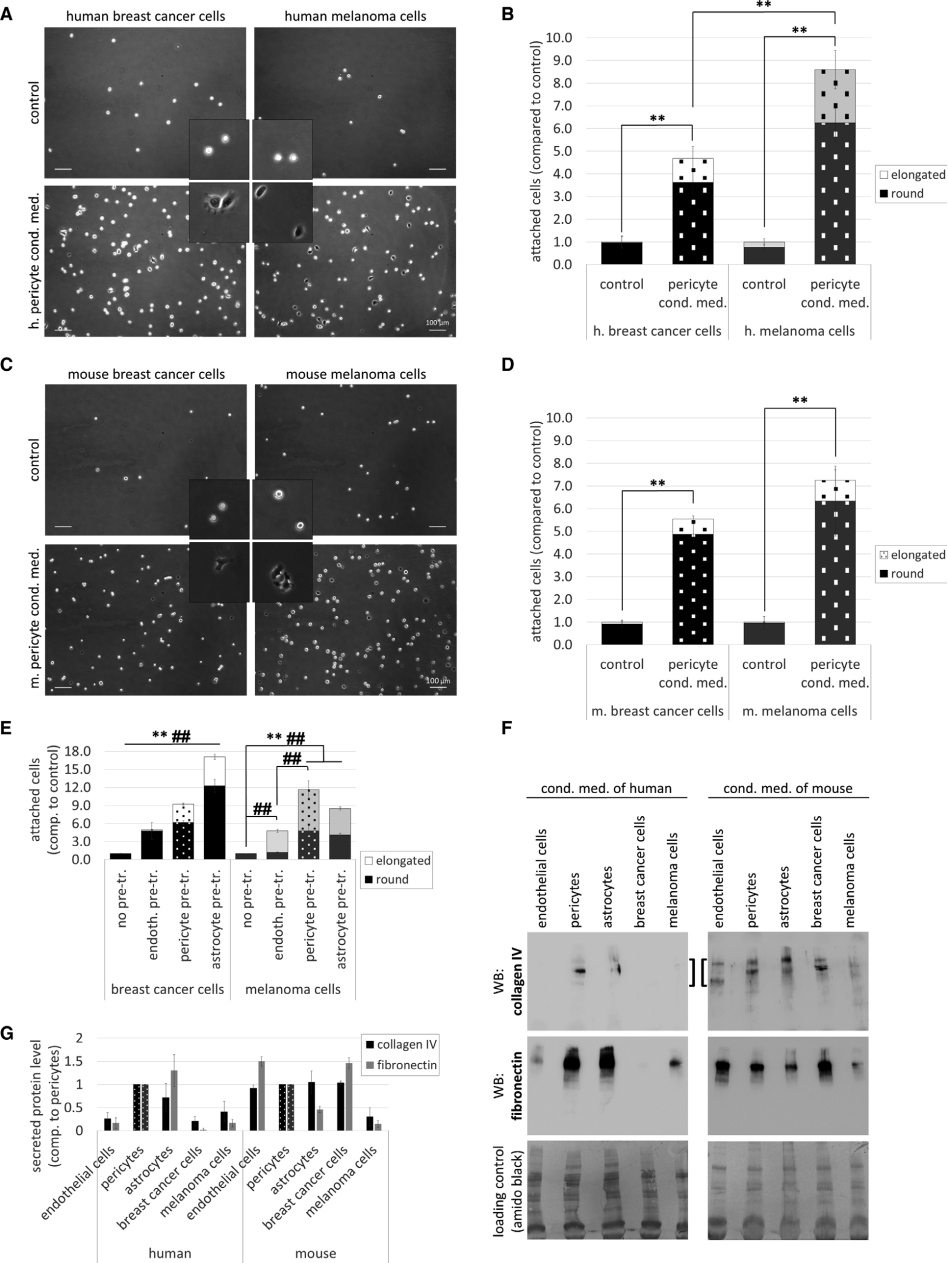
Role of pericyte‐secreted factors in tumor cell adhesion. (A) Representative phase‐contrast images of human breast cancer or melanoma cells (MDA or A2058, respectively) attached to noncoated cell culture dishes in control or pericyte‐conditioned media after 120 min (MDA) or 20 min (A2058). Scale bar = 100 µm. (B) Quantification of adhesion results shown in (A). *N* = 5, average ± SD, **P < 0.01 (ANOVA and Bonferroni's *post hoc* test). (C) Representative phase‐contrast images of mouse breast cancer or melanoma cell adhesion (4T1 and B16 cells, respectively) 20 min after plating. Scale bar = 100 µm. (D) Quantification of adhesion results shown in (C). *N* = 5, average ± SD, **P < 0.01 (ANOVA and Bonferroni's *post hoc* test). Insets in (A) and (C) show cells having typical round and elongated morphology (upper and bottom panels, respectively) (2.5 times of magnification compared to whole pictures). (E) Adhesion of MDA and A2058 cells in culture dishes pretreated for 24 h with conditioned media of D3, HBVP, or HA cells. *N* = 3, average ± SD, **P < 0.01 (all cells), ^##^ *P* < 0.01 (elongated cells). Breast cancer cells: significant difference among all groups (ANOVA and Bonferroni's *post hoc* test). (F) Secretion of collagen type IV and fibronectin into culture media of brain and tumor cells (representative western blot images). (G) Quantification of collagen type IV and fibronectin western blots. *N* = 3, average ± SD (statistically not analyzed).

First, we seeded breast cancer and melanoma cells in control and pericyte‐conditioned media and performed an adhesion assay. As shown in Fig. 3A–D, both human and mouse breast cancer and also melanoma cells attached to the culture dish surface and elongated more rapidly in pericyte‐conditioned media, than in control conditions. The difference between cells seeded in control and conditioned media was very high and significant (Fig. 3C,D). Next, prior to seeding of the tumor cells, we pretreated the culture dishes with conditioned media of brain pericytes, endothelial cells, or astrocytes and performed an adhesion assay. Pericyte‐conditioned medium enhanced attachment of both breast cancer and melanoma cells more effectively than endothelial‐derived medium, while astrocyte‐secreted factors had a more pronounced effect on breast cancer cells and were less effective on the adhesion of melanoma cells (Fig. 3E).

Adhesive substrate for the cells is provided by extracellular matrix, for example, basement membrane proteins. Therefore, we tested secretion of type IV collagen and fibronectin, two major components of the vascular basal lamina in the brain. According to our data, pericytes—similar to astrocytes—secreted high amounts of collagen type IV and fibronectin (Fig. 3F,G). These results explain the prominent adhesion enhancing effect of pericytes and astrocytes.

Cells attach to the extracellular matrix in focal adhesions, which are regulated by FAK (focal adhesion kinase), Src, and other signaling proteins [22]. Both FAK and Src phosphorylation increased in breast cancer and melanoma cells seeded in pericyte‐conditioned media (Fig. S2A,B). In addition, PP2, a specific Src inhibitor—but not the structurally related negative control PP3—significantly reduced the adhesion‐inducing effect of pericyte‐conditioned media (Fig. S2C–E).

### 3.3 Effects of pericytes on tumor cell proliferation

**Fig. 4:**
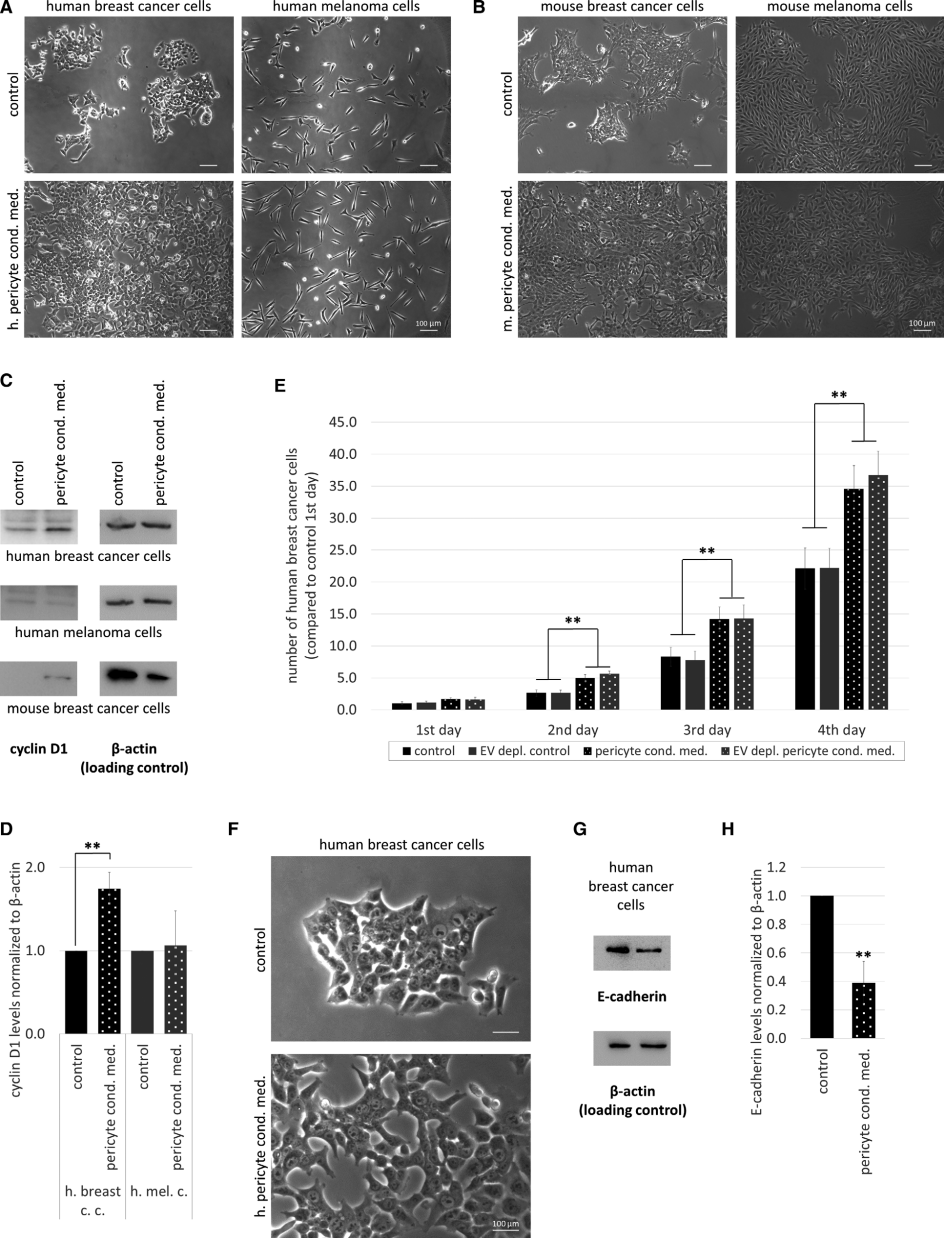
Role of pericyte‐secreted factors in tumor cell proliferation. (A, B) Representative phase‐contrast images of tumor cells grown in control or pericyte‐conditioned medium for 4 days. Scale bars = 100 µm. (C) Cyclin D1 expression in tumor cells grown in control or pericyte‐conditioned medium for 4 days. (D) Quantification of cyclin D1 western blots. *N* = 3, average ± SD, **P < 0.01 (ANOVA and Bonferroni's *post hoc* test). (E) Quantification of cell numbers of MDA cells grown in control, pericyte‐conditioned, and EV‐depleted media. *N* = 4, average ± SD, **P < 0.01 (ANOVA and Bonferroni's *post hoc* test). (F) Representative high‐magnification phase‐contrast images of MDA cells grown in control or pericyte‐conditioned medium for 4 days. Scale bar = 100 µm. (G) E‐cadherin expression in MDA cells grown in control or pericyte‐conditioned medium for 4 days. (H) Quantification of E‐cadherin western blots. *N* = 3, average ± SD, **P < 0.01 (Student's *t*‐test).

As a next step, we aimed to understand how brain pericytes influence tumor growth. Therefore, we performed a tumor cell proliferation assay in the presence and absence of factors released by pericytes. Four days after plating, the number of breast cancer cells was substantially higher in pericyte‐conditioned than in control media (Fig. 4A,B). This was clearly observed both in the human and in the mouse model. On the other hand, melanoma cells did not respond with increased proliferation to the presence of pericyte‐secreted factors (Fig. 4A and Fig. S3A). In parallel, there was a significant increase in the expression of cyclin D1 in human and mouse breast cancer cells, but not in melanoma cells cultured in pericyte‐conditioned media (Fig. 4C,D).

Besides soluble factors, conditioned media may contain EVs, like microvesicles and exosomes. In order to distinguish between effects of EVs and soluble factors, we prepared EV‐depleted conditioned medium and compared it with complete conditioned medium in a proliferation assay. EV depletion did not influence the proliferation‐increasing effect of pericyte‐conditioned medium (Fig. 4E); therefore, soluble factors released by pericytes are responsible for the observed phenomenon.

Interestingly, breast cancer cells in the conditioned media, although higher in number, appeared to have fewer contact points with each other and became more dissociated (Fig. 4F). In line with this, E‐cadherin expression decreased significantly in breast cancer cells being exposed to pericyte‐secreted factors (Fig. 4G,H).

### 3.4 Insulin‐like growth factor expression in cerebral pericytes

**Fig. 5:**
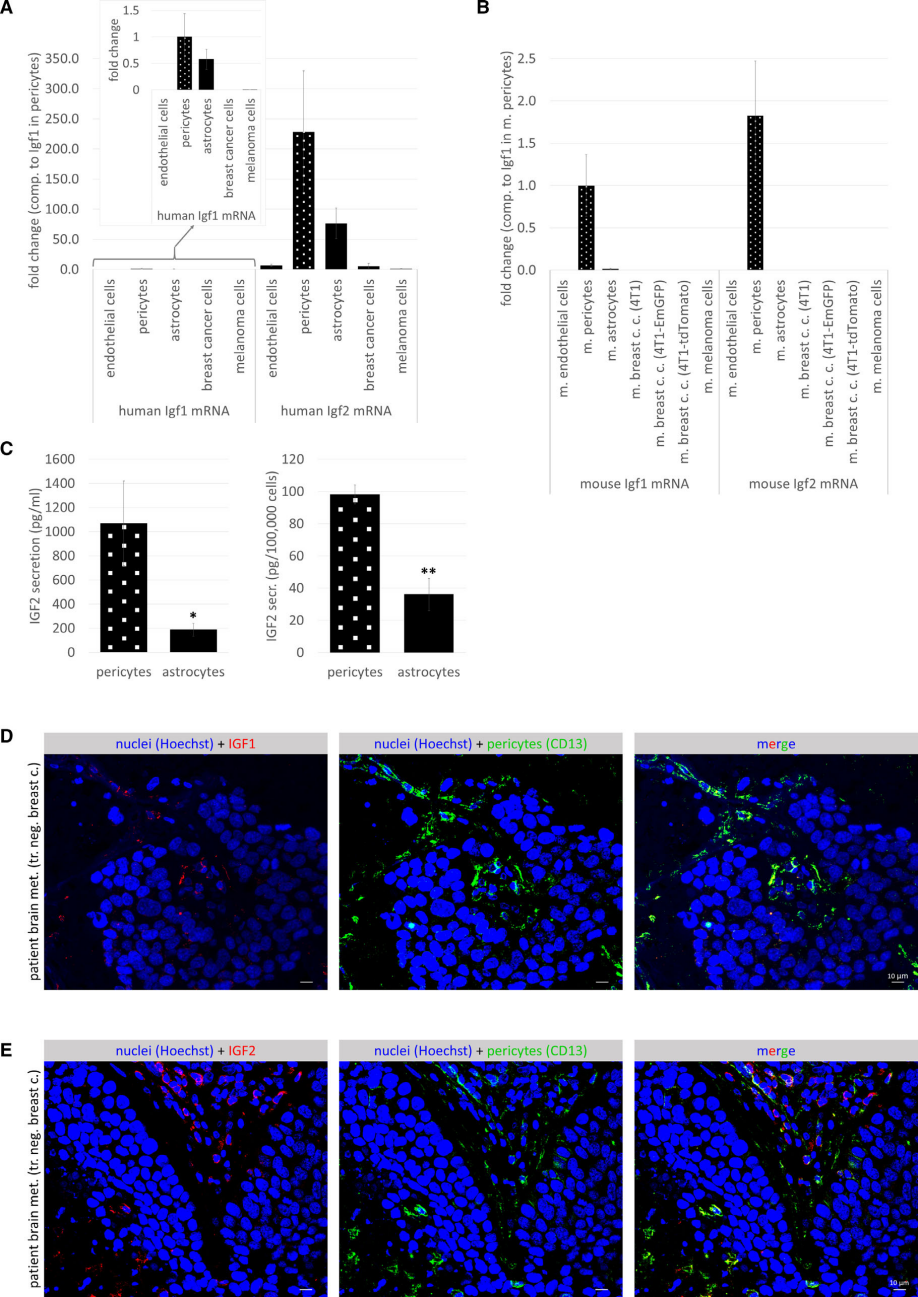
Insulin‐like growth factor expression in pericytes. (A, B) Igf1 and Igf2 mRNA expression in human and mouse brain and tumor cells, as assessed by qPCR with GAPDH as housekeeping gene. Results are shown as fold change in comparison with Igf1 in pericytes. Inset in (A) shows human Igf1 expression in a lower scale range. *N* = 3, average ± SD (statistically not analyzed). (C) IGF2 secretion in human brain pericytes and astrocytes, as assessed by ELISA. *N* = 3 pericytes, *N* = 2 astrocytes, average ± SD, *P < 0.05, **P < 0.01 (Student's *t*‐test). (D) Representative immunofluorescence staining showing co‐localization of IGF1 and CD13 in human TNBC brain metastatic tissue. Scale bar = 10 µm. (E) Representative immunofluorescence staining showing co‐localization of IGF2 and CD13 in human TNBC brain metastatic tissue. Scale bar = 10 µm.

In order to identify which cerebral pericyte‐secreted factors might be involved in enhancing tumor cell proliferation, we first performed a database search. In the http://betsholtzlab.org/VascularSingleCells/database.html collection [23], we found Igf2 mRNA having the highest expression in pericytes among cells of the NVU. Igf2 mRNA expression level was 487.25 times higher in pericytes than in endothelial cells, and 86.62 times more in pericytes vs. astrocytes in the mouse brain (Fig. S3B). In our human and mouse pericytes, not only Igf2, but Igf1 mRNA was also expressed (Fig. 5A,B). More Igf2 than Igf1 mRNA was found both in human and mouse pericytes; however, the ratio was higher in human cells. Mouse astrocytes expressed very low amounts of Igf1 and Igf2 mRNA, while in HAs both Igf1 and Igf2 mRNA levels were higher than in tumor cells, but still lower than in pericytes.

These data were confirmed on the protein level using ELISA. Human pericytes secreted more than 1000 pg·mL^−1^ IGF2 corresponding to almost 100 pg/100 000 cells, while in astrocytes we detected significantly less IGF2 (< 200 pg·mL^−1^, < 40 pg/100 000 cells) (Fig. 5C). IGF1 levels in both pericyte‐ and astrocyte‐conditioned media were below the detection limit of 1.95 ng·mL^−1^. By using immunofluorescence, we found both IGF1 and IGF2 to be expressed in CD13‐positive pericytes in the human brain (Fig. S3C,D). IGF1 was also detected in a few CD13‐negative cells, probably astrocyte end‐feet, while IGF2 was mainly expressed in pericytes. In human TNBC brain metastatic lesions, the expression of IGF1 and IGF2 was highly restricted to perivascular cells (Fig. 5D,E).

### 3.5 Role of brain pericyte‐secreted IGFs in proliferation of TNBC cells

**Fig. 6:**
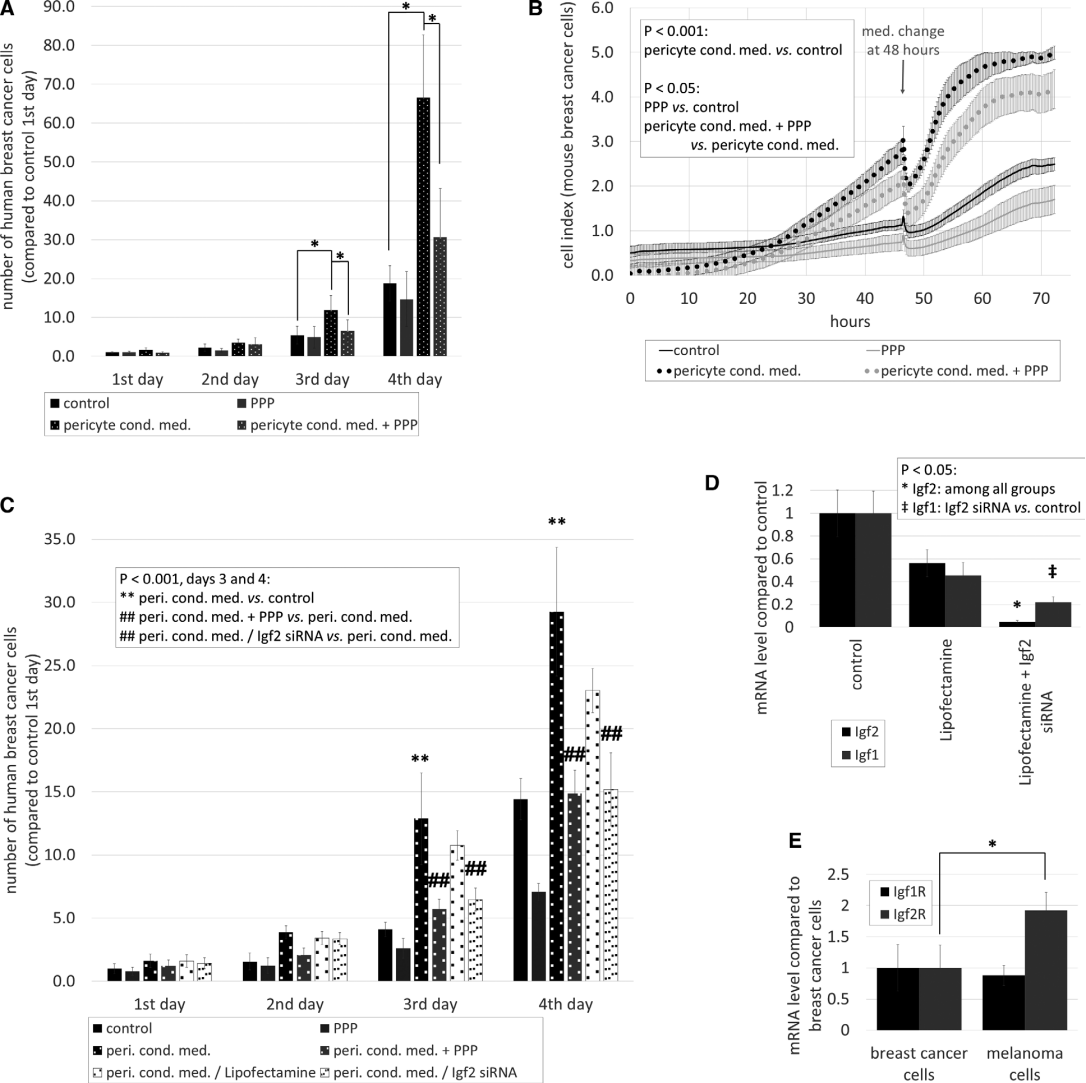
Role of IGFs in breast cancer cell proliferation *in vitro*. (A) Quantification of MDA cell growth in control or pericyte‐conditioned media in the presence or absence of PPP. Cells were counted in phase‐contrast micrographs. *N* = 5, average ± SD, *P < 0.05 (ANOVA and Bonferroni's *post hoc* test). (B) Growth of 4T1 cells in control or pericyte‐conditioned media in the presence or absence of PPP, as assessed by impedance measurements. *N* = 4, average ± SD, *P* < 0.001 (cells cultured in pericyte‐conditioned media compared to control), *P* < 0.05 (cells treated with PPP compared to control; cells cultured in pericyte‐conditioned media and PPP compared to cells cultured in pericyte‐conditioned media) (two‐way ANOVA with repeated measures and Tukey's *post hoc* test). (C) Growth of MDA cells cultured in control or pericyte‐conditioned media in the presence or absence of PPP, or in conditioned media of Lipofectamine‐treated or Igf2‐silenced pericytes. *N* = 5, average ± SD, **P < 0.01 (compared to control), ^##^ *P* < 0.01 (compared to cells cultured in pericyte‐conditioned media) (ANOVA and Bonferroni's *post hoc* test). (D) Effect of Igf2 silencing on Igf2 and Igf1 mRNA expression in HBVP cells, as assessed by qPCR with GAPDH as housekeeping gene. *N* = 3, average ± SD, *P* < 0.05 (ANOVA and Bonferroni's *post hoc* test). (E) Igf1R and Igf2R mRNA expression in human tumor cells. Results are shown as fold change in comparison with Igf1R in breast cancer cells. *N* = 3, average ± SD, *P < 0.05 (ANOVA and Bonferroni's *post hoc* test).

By binding to the type 1 insulin‐like growth factor receptor (IGF1R), IGFs are involved in growth and survival of both normal and neoplastic cells [24]. Therefore, we next tested whether pericyte‐secreted IGFs are responsible for the increased proliferation rate of breast cancer cells. For this purpose, we used a selective inhibitor of IGF1R, PPP, which efficiently blocks IGF1R without inhibiting the insulin receptor, and has low toxicity in rodents [25]. Addition of PPP to pericyte‐conditioned medium reduced its proliferation‐inducing effect to almost control levels both in human and mouse breast cancer cells (Fig. 6A,B).

In addition, we silenced Igf2 gene in pericytes, collected conditioned media, and performed a proliferation assay (Fig. 6C). Igf2 silencing was very efficient, and although slightly affected Igf1 mRNA expression as well (Fig. 6D), it can be considered specific, due to the much higher expression of IGF2 compared to IGF1 in brain pericytes (Fig. 5). Proliferation rate of breast cancer cells in conditioned media of Igf2‐silenced pericytes was similar to control cells, or cells cultured in pericyte‐conditioned media in the presence of PPP (Fig. 6C).

In line with secreted IGF2 levels, astrocyte‐conditioned media had a much less pronounced effect on proliferation of breast cancer cells and no effect on melanoma cell division. PPP reversed the astrocyte‐conditioned medium‐induced slight increase in breast cancer cell proliferation (Fig. S4A,B).

In contrast to breast cancer cells, proliferation of melanoma cells was not affected by PPP (Fig. S4C). In order to understand the discrepancy between the effect of pericyte‐derived IGFs on breast cancer and melanoma cells, we analyzed receptor expression level of the two cell types. Igf1R mRNA expression level was similar in the two cell types; however, melanoma cells expressed almost two times more Igf2R mRNA than breast cancer cells (Fig. 6E). This is in line with data from the Human Protein Atlas (https://www.proteinatlas.org/ENSG00000197081‐IGF2R/pathology), which indicates a higher average mRNA and protein expression of IGF2R in melanoma than in breast cancer. This might partly explain the resistance of melanoma cells to IGFs, because IGF2R directs IGF2 to lysosomes to attenuate signaling [26].

**Fig. 7:**
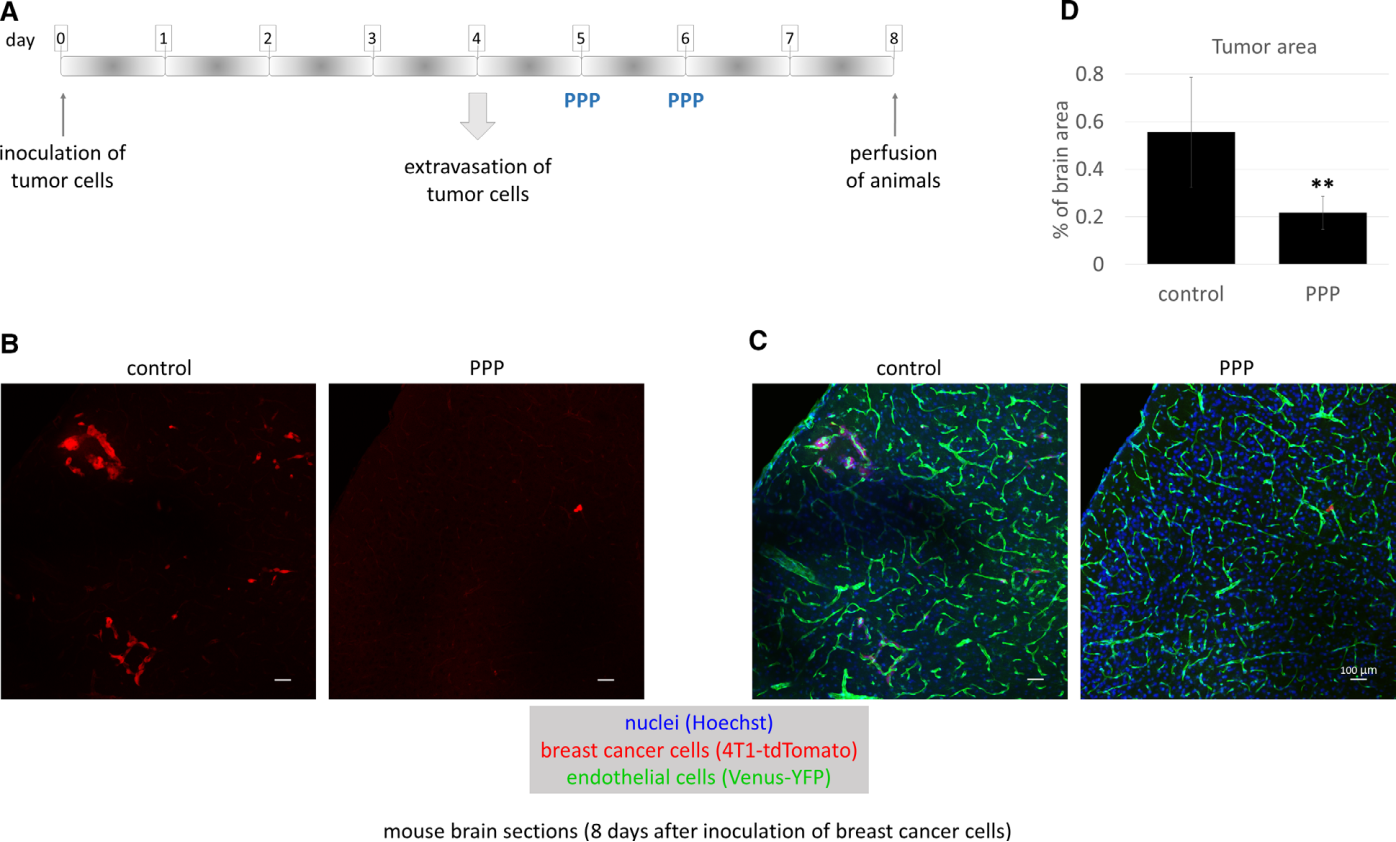
Role of IGFs in breast cancer cell proliferation *in vivo*. (A) Experimental design of studying the role of IGFs in TNBC proliferation in the brains of mice. (B, C) Representative confocal micrographs of parietal brain sections of control FVB/Ant:TgCAG‐yfp mice and animals treated with PPP, 8 days after inoculation of 4T1‐tdTomato cells. Red channel represents tumor cells (B). In (C), the same sections are shown as in (B) in three channels: red, green (endothelium), and blue (nuclei) channels. Scale bar = 100 µm. (D) Quantification of tumor size in control and PPP‐treated mice. Tumor area is presented as percentage of brain area, as calculated from 17 brain sections of three animals/treatment. Average ± SEM, **P < 0.01 (two‐way ANOVA without replication).

Since PPP can cross the BBB [27], we next designed an *in vivo* setup to test the role of IGFs in breast cancer brain metastasis development. In order to test metastatic cell proliferation in the brain parenchyma, PPP was administered on days 5 and 6 after inoculation of tumor cells (Fig. 7A), when majority of cells have completed extravasation from cerebral capillaries [16]. Our results clearly showed that PPP inhibited proliferation of breast cancer cells in the brains of mice (Fig. 7B,C). The brain area covered by tumor cells became significantly, more than 2.5 times smaller in animals treated with the IGF1R inhibitor (Fig. 7D).

## 4 Discussion

Implicated in extravasation of malignant cells through the BBB and influencing their ability to survive and proliferate in the cerebral environment, cells of the NVU have been increasingly recognized as key players in brain metastasis formation. Although interactions between metastatic cells and CECs, astrocytes, or microglia are relatively well characterized [6], our knowledge about the role of pericytes is restricted to indirect effects on tumor cells, including regulation of blood–tumor barrier permeability [28] and secretion of connective tissue proteins [21]. By remodeling the perivascular niche [29], increasing angiogenesis [29], and acquiring an immunosuppressive function [31, 32], pericytes have undisputed roles in promoting primary brain tumors. Therefore, we hypothesized that pericytes might facilitate progression of brain metastases as well.

By using *in vivo* and *in vitro* techniques and human samples, here we show and characterize direct interactions of brain pericytes with metastatic cells. First, others [20] and we have observed that pericytes remain attached to the vessels and are retained in metastatic lesions, while astrocytes are expelled to the border of the tumor [16]. In addition, inside the tumor mass, we found PDGFRβ and CD13 double‐positive, nonperivascular pericyte‐like cells, origin of which is unknown. Second, we proved direct interaction between tumor cells and pericytes *in vitro* as well, by showing that breast cancer cells preferentially attach to pericytes, avoiding free culture surfaces.

Our results point to a pronounced role of pericyte‐secreted soluble factors, having chemoattractant, adhesion‐ and proliferation‐enhancing effects mainly on triple‐negative mammary carcinoma cells. We observed that pericytes secrete proteins of the extracellular matrix to enhance formation of tumor cell focal adhesions. In the brain, interaction with the vascular basement membrane is critical for the survival of metastatic cells [33, 34]. Embedded in the inner, endothelial layer of the vascular basal lamina, pericytes substantially contribute to the secretion of matrix proteins [35], which might be particularly important in metastatic tumors, which tend to detach astrocytes—another major source of basal membrane proteins—from co‐opted vessels. This is in line with accumulation of collagen secreted by perivascular cells observed in papillary‐type human brain metastases [21].

Besides enhancing adhesion to the substrate, pericytes inhibit intercellular adhesions and expression of E‐cadherin, conferring breast cancer cells a migratory, invasive phenotype, characteristic of cells undergoing epithelial‐to‐mesenchymal transition (EMT) [36]. In line with these results, pericytes have been shown to be major sources of EMT factors in glioma [37]. We have also found that pericytes have a prompt effect on the proliferation of TNBC, but not of melanoma cells. Bioinformatics analysis, followed by experimental approaches confirmed high expression of IGFs in pericytes, contributing to breast cancer cell proliferation *in vitro* and *in vivo*. Indeed, in addition to regulating cell division, the IGF system has also been shown to promote induction of the EMT phenotype in epithelial tumors [38, 39] and to play an important role in the maintenance of cancer stem cells in breast cancer [40].

Insulin‐like growth factor signaling has been implicated in the growth and survival of both normal and malignant cells. IGF1R expression and activity increase in several tumor types, including breast cancer [41], resulting in enhanced proliferation rate, metastatic capacity, and resistance to chemotherapy [42]. Therefore, targeting of the IGF axis has been in the focus of therapeutic approach developments in numerous malignant diseases [43]. However, since many clinical trials have failed and further studies have been discontinued due to toxicity or low efficacy of the tested compounds, identification of predictive biomarkers is urged to define potentially responsive patient subgroups. Nevertheless, brain metastatic disease has not been specifically investigated in IGF1‐targeting clinical trials, although inhibition of IGF1R has been shown to reduce breast cancer brain metastasis development in experimental models [44]. Moreover, a clinical phase I study has been completed with PPP (AXL1717) in patients with primary brain tumors, showing that the drug was well tolerated and no major side effects occurred [45].

## 5 Conclusions

Taken together, our results show that pericytes play crucial role in the development of brain secondary tumors by directly influencing key steps of metastatic colonization of the CNS. Secretion of soluble factors, including extracellular matrix proteins and growth factors, endows brain pericytes with significant pro‐metastatic features, especially in breast cancer. Therefore, influencing pericyte functions might represent a future therapeutic opportunity in brain metastatic disease. This is in line with emerging approaches, which tend to target the more stable tumor microenvironment in addition to the very plastic cancer cells. Moreover, our study underlines the importance of IGF axis inhibition as a potential strategy in brain metastases, especially as there exists a compound (PPP/AXL1717) with high selectivity on IGF1R that not only has low toxicity, but is also BBB permeable. This is a great advantage, since the BBB represents the highest obstacle in the development of drugs targeting the CNS [46].

## Conflict of interest

The authors declare no conflict of interest.

## Author contributions

KM, IAK, and IW designed research study; KM, ÁM, CF, MK, FG, ZR, AEF, ÁN‐T, and JH performed research; KM, LT, IAK, and IW analyzed the data; IW and IAK supervised research; IW drafted the manuscript; all authors approved final version.

## Supporting information


**Fig. S1.** Tumor cell adhesion onto brain cells.
**Fig. S2.** Signaling pathways involved in pericyte‐enhanced tumor cell adhesion.
**Fig. S3.** Tumor cell proliferation and IGF expression in the brain.
**Fig. S4.** Effect of IGF inhibition on tumor cell proliferation.
**Table S1.** Primary antibodies used for immunofluorescence (IF) and western blot (WB).
**Table S2.** Primers used for real‐time PCR.
