# Supplementary material for: Pericyte‐secreted IGF2 promotes breast cancer brain metastasis formation
Source: Mol Oncol. 2020 Jun 26;14(9):2040–57. doi: 10.1002/1878-0261.12752 (PMC7463359; doi:10.1002/1878-0261.12752)
Supplement: Supplementary file 1 — Fig. S1. Tumor cell adhesion onto brain cells. Fig. S2. Signaling pathways involved in pericyte‐enhanced tumor cell adhesion. Fig. S3. Tumor cell proliferation and IGF expression in the brain. Fig. S4. Effect of IGF inhibition on tumor cell proliferation. Table S1. Primary antibodies used for immunofluorescence (IF) and western blot (WB). Table S2. Primers used for real‐time PCR. [file MOL2-14-2040-s001.docx]

Supplementary files

# Supplementary tables

## **Supplementary Table 1.** Primary antibodies used for immunofluorescence (IF) and western-blot (WB).

| **Primary antibody** | **Catalog number** | **Dilution** |
| --- | --- | --- |
| goat polyclonal anti-**CD13** | AF2335 (BioTechne, Minneapolis, MN, USA) | 1:150 IF |
| rabbit monoclonal anti-**PDGFRβ** | 3169 (Cell Signaling Technology, Boston, MA, USA) | 1:100 IF |
| rabbit polyclonal anti-**IGF1** | ab9572 (Abcam, Cambridge, UK) | 1:100 IF |
| rabbit polyclonal anti-**IGF2** | ab226989 (Abcam) | 1:100 IF |
| m. monoclonal anti-**cytokeratin** | MA5-12231 (Thermo Fisher Scientific) | 1:150 IF |
| mouse monoclonal anti-**β-actin** | sc-47778 (Santa Cruz Biotechnology, Santa Cruz, CA, USA) | 1:1000 WB |
| rabbit polyclonal anti-**fibronectin** | ab2413 (Abcam) | 1:1000 WB |
| rabbit polyclonal anti-**collagen IV** | ab6586 (Abcam) | 1:1000 WB |
| mouse monoclonal anti-**cyclin D1** | sc-8396 (Santa Cruz Biotechnology) | 1:500 WB |
| m. monoclonal anti-**E cadherin** | C20820 (BD Transduction Lab., San Diego, CA, USA) | 1:250 WB |
| rabbit polyclonal anti-**FAK** | 3285 (Cell Signaling Technology) | 1:500 WB |
| rabbit polyclonal anti-**pFAK** | 3283 (Cell Signaling Technology) | 1:500 WB |
| mouse monoclonal anti-**Src** | 2110 (Cell Signaling Technology) | 1:250 WB |
| rabbit polyclonal anti-**pSrc** | 2101 (Cell Signaling Technology) | 1:250 WB |

## **Supplementary Table 2.** Primers used for real-time PCR.

| **Gene** | **Forward primer (5’=>3’)** | **Reverse primer (5’=>3’)** |
| --- | --- | --- |
| human IGF1 | AGAGCCTGCGCAATGGAATA | GAGATGCGAGGAGGACATGG |
| human IGF2 | GACCGCGGCTTCTACTTCA | GGGGTATCTGGGGAAGTTGT |
| human IGFR1 | GGCACAATTACTGCTCCAAAGAC | CAAGGCCCTTTCTCCCCAC |
| human IGFR2 | AGCGAGAGCCAAGTGAACTC | TCGCTGTAAGCAGCTGTGAA |
| mouse IGF1 | TGCTAAATCTCACTGTCACTGCT | CAGAGCGCCAGGTAGAAGAG |
| mouse IGF2 | CCCCAGCCCTAAGATACCCT | CACCAACATCGACTTCCCCA |
| GAPDH | GTGAAGGTCGGTGTCAACG | GTGAAGACGCCAGTAGACTC |

# Supplementary figures


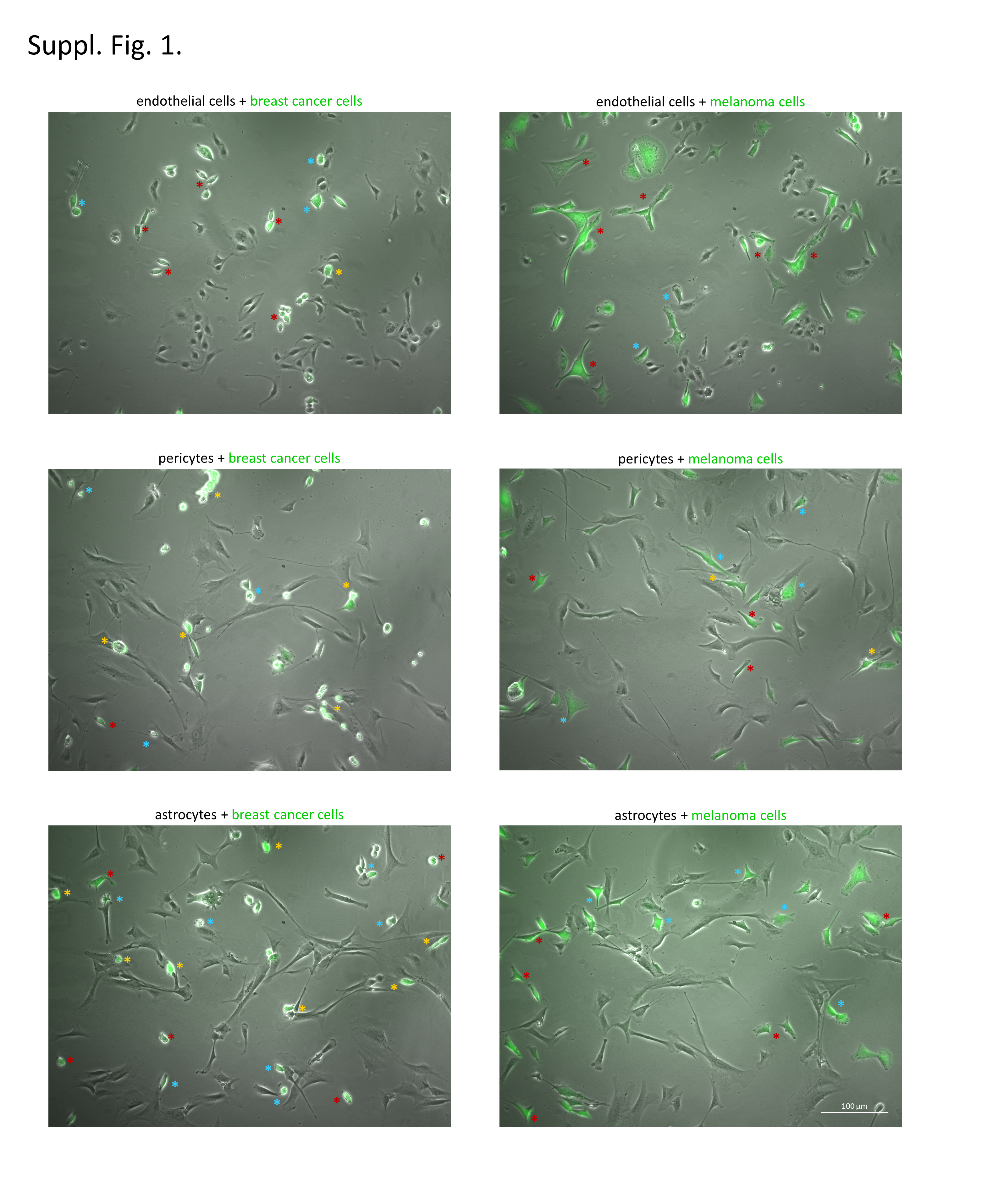


### Supplementary Fig. 1. Tumor cell adhesion onto brain cells.

Representative images of human breast cancer cells (MDA-GFP) and human melanoma cells (A2058-GFP) seeded onto human brain endothelial (D3), pericyte (HBVP) or astrocyte (HA) cultures. Red asterisks mark tumor cells attached to free dish surfaces, cyan asterisks indicate tumor cells attached nearby brain cells, yellow asterisks show tumor cells attached onto brain cells. Quantification is shown in Fig. 1 E.


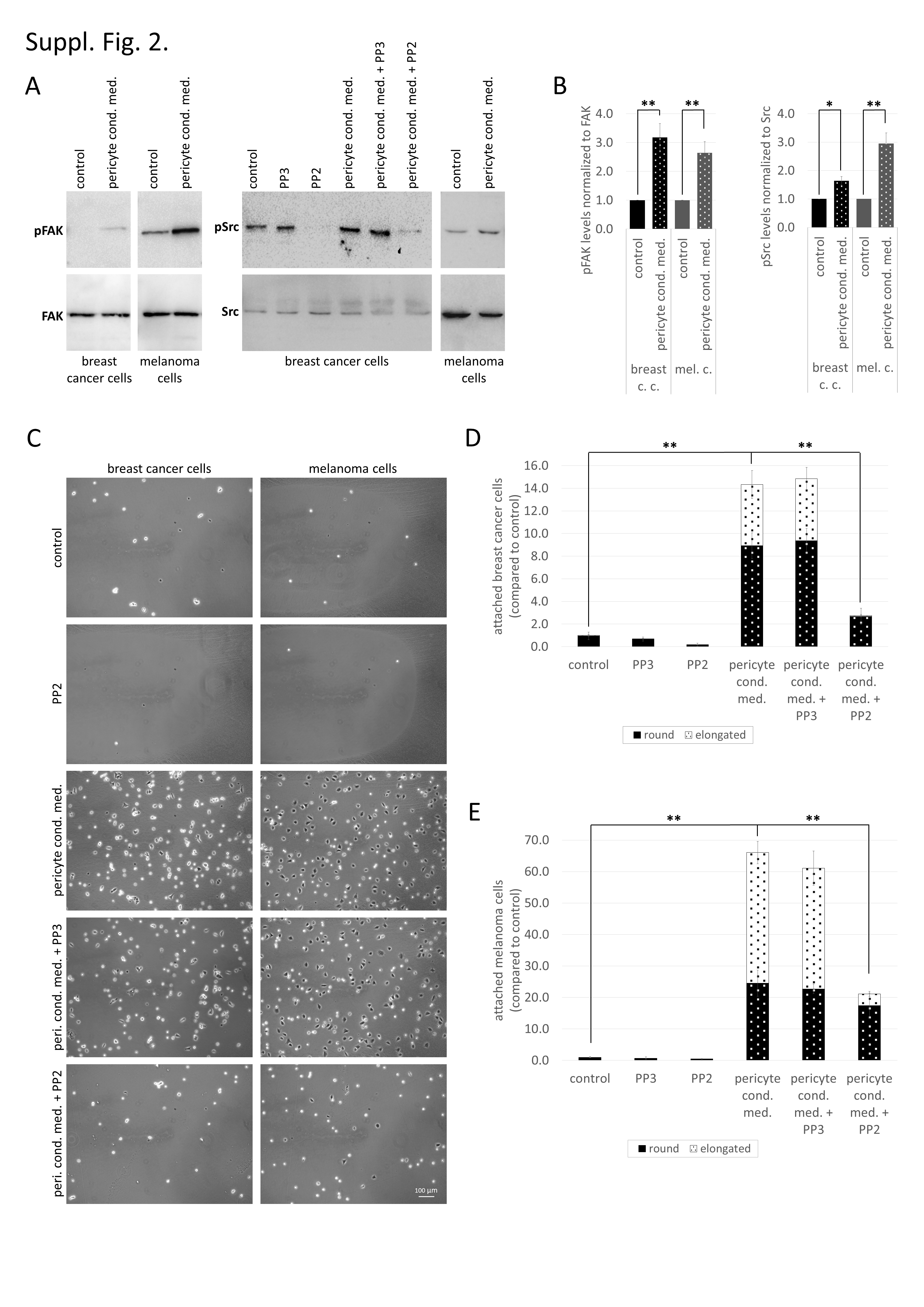


### Supplementary Fig. 2. Signaling pathways involved in pericyte-enhanced tumor cell adhesion.

**A**: Representative western-blots showing FAK and Src phosphorylation in MDA and A2058 cells cultured in HBVP-conditioned media, 120 minutes and 20 minutes after seeding, respectively. **B**: Quantification of blots shown in (A). N = 2, N = 3, average +/- SD, * P < 0.05.* P < 0.05, ** P < 0.01. **C**: Representative phase-contrast images of MDA and A2058 cells plated in control or HBVP-conditioned media in the presence or absence of the Src kinase inhibitor PP2 (10 µM) or its negative control PP3 (10 µM). **D**, **E**: Quantification of data shown in (C). N = 3, average +/- SD, * P < 0.05, ** P < 0.01.


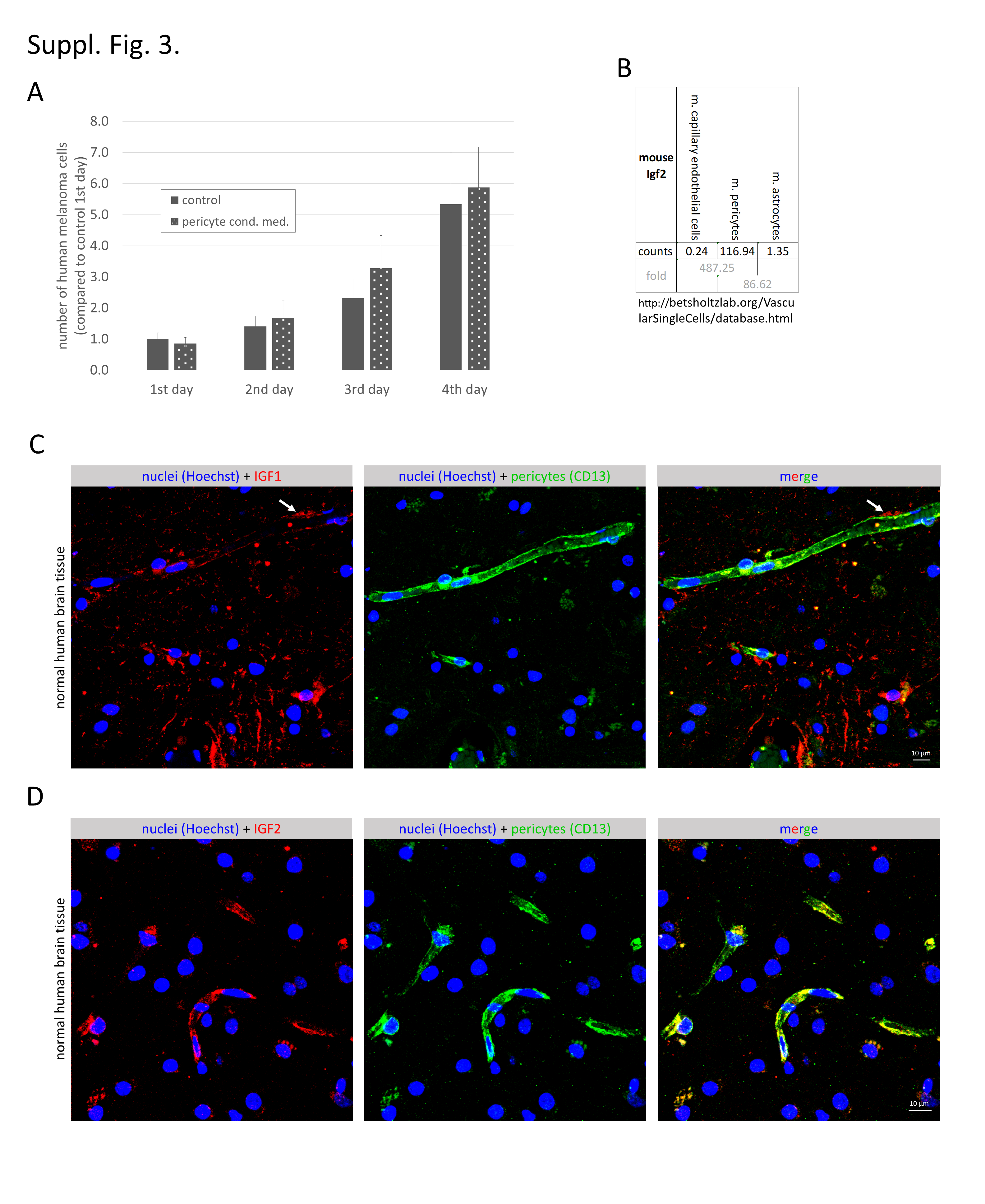


### Supplementary Fig. 3. Tumor cell proliferation and IGF expression in the brain.

**A**: Quantification of proliferation assay in A2058 cells. N = 3, average +/- SD **B**: Igf2 mRNA expression in brain pericytes (data from betsholtzlab.org database). **C**, **D**: IGF1 and IGF2 expression in normal human brain tissue. White arrow indicates an IGF1-positive, but CD13-negative astrocyte end-feet-like structure.


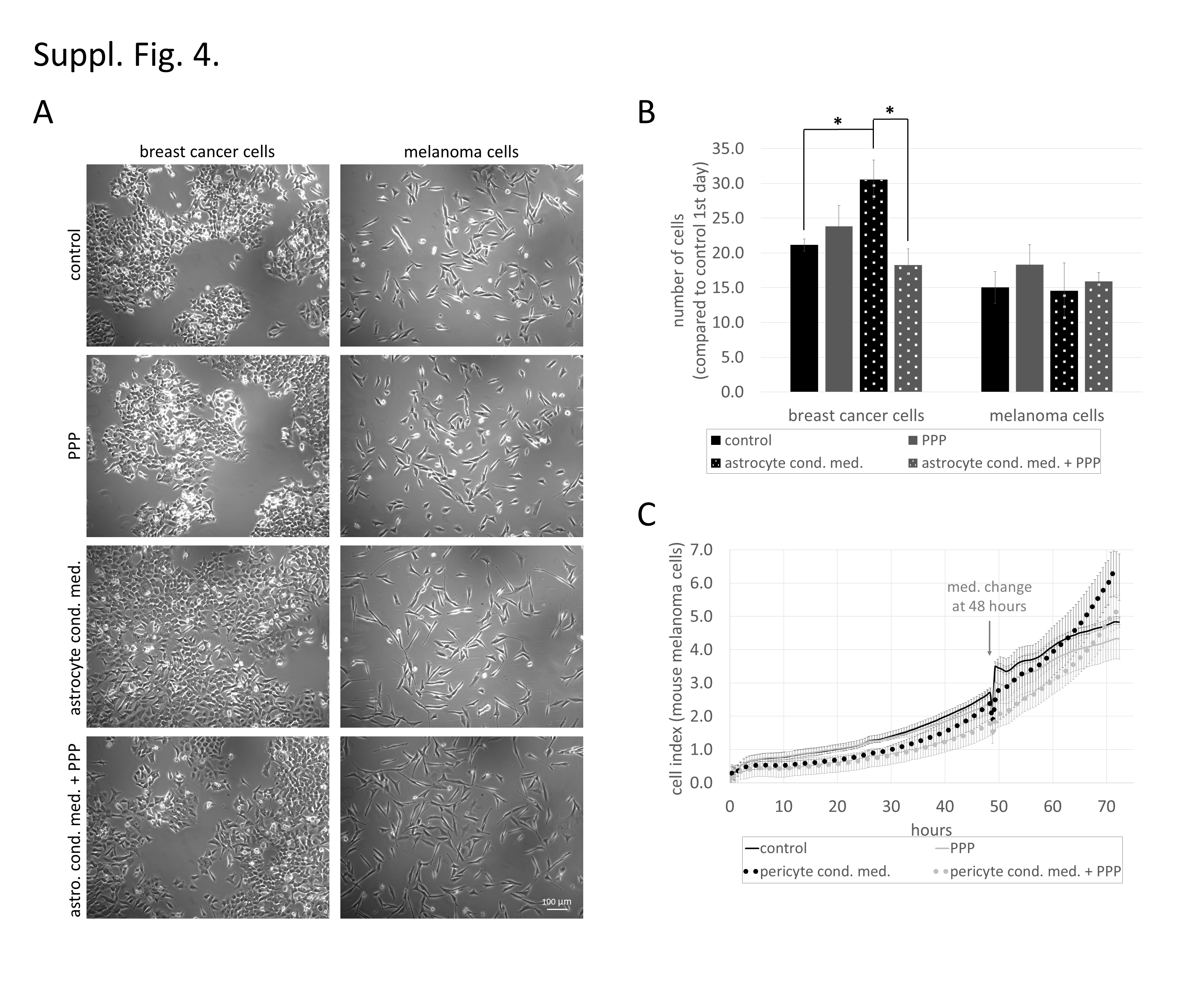


### Supplementary fig. 4. Effect of IGF inhibition on tumor cell proliferation.

**A**: Representative phase-contrast images of MDA and A2058 cells grown for 4 days in control or HA-conditioned media, in the presence or absence of PPP. **B**: Quantification of data shown in (A). N = 3, average +/- SD, * P < 0.05. **C**: Growth of B16 cells in control or mouse pericyte-conditioned media in the presence or absence of PPP, as assessed by impedance measurements. N = 3, average +/- SD.
